# Does Wearing a Face Mask During the COVID-19 Pandemic Increase the Incidence of Dermatological Conditions in Health Care Workers? Narrative Literature Review

**DOI:** 10.2196/22789

**Published:** 2021-05-06

**Authors:** Robyn-Jenia Wilcha

**Affiliations:** 1 Faculty of Biology, Medicine and Health University of Manchester Manchester United Kingdom

**Keywords:** COVID-19, dermatology, face masks, health care worker, incidence, literature, mask, N95 mask, review, skin

## Abstract

**Background:**

COVID-19 is a health emergency. SARS-CoV-2 was discovered in Wuhan (Hubei Province, China) and has rapidly spread worldwide, leaving no country untouched. COVID-19 is a respiratory infection characterized by a pneumonia of unknown etiology. It is transmitted through respiratory droplets; for example: through breathing, talking, and coughing. Transmission of the virus is high. Health care workers play important roles in helping those affected by COVID-19; this could not be done without the use of personal protective equipment (PPE). PPE involves the use of goggles, masks, gloves, and gowns and is known to reduce COVID-19 transmission; however, multiple reports of skin disease and damage associated with occupational mask-wearing have emerged.

**Objective:**

The objective of this study is to review the literature for newly emerging dermatological conditions as a result of occupational mask-wearing during the COVID-19 pandemic.

**Methods:**

A narrative review of new reports of dermatological conditions associated with occupational mask-wearing was carried out in May 2020 by referencing keywords including: “covid mask dermatology,” “covid dermatological damage,” “covid mask skin,” “covid N95 mask damage,” and “covid mask skin damage” from PubMed, supplemented by searches on both Google Scholar and ResearchGate. A total of 287 articles were found, of which 40 were successfully included in this study, and an additional 7 were selected from the reference lists of these 40 articles. The findings were tabulated and analyzed under the following headings: dermatological diagnosis, causes, and management.

**Results:**

Qualitative analysis of the reviewed data was carried out. A number of dermatological conditions were found to increasingly occur owing to prolonged and frequent use of face masks. Pressure-related injuries were often the most serious complaint; recommendations to reduce this type of injury include the use of hydrocolloid dressings, plastic handles, education, and regular moisturization. Innovation in PPE as well as services, such as virtual clinics, need to be advanced to protect the welfare of health care staff.

**Conclusions:**

In these unprecedented times, PPE has been an effective barrier to the transmission of COVID-19 among health care workers. This has allowed health care workers to provide care to patients, with minimal risk. However, our findings suggest that despite the obvious benefits of using face masks to protect the respiratory system, there are also considerable health consequences to the skin. Future research studies are required to focus on improving face masks to ensure both the protection of the respiratory system as well as skin care, which, according to our study, has been overlooked.

## Introduction

COVID-19, formerly known as the novel coronavirus infection, is a global public health emergency [1]. The causative virus was initially detected in Wuhan (Hubei Province, China) in December 2019; bats have subsequently been linked to the spread of the disease [1]. Typical symptoms of COVID-19 prominently include fever, cough, sore throat, breathlessness, fatigue, headache, and changes to cognition, although some infected individuals may be asymptomatic [1]. Human-to-human transmission of the virus occurs at high rates, and the virus can be spread through direct contact and respiratory particles [2]. Respiratory particles may be transmitted through breathing, talking, coughing, and sneezing [2].

As of May 2020, no drug nor antiviral vaccine has been officially approved for the treatment of COVID-19 [2]. The current state of emergency due to the COVID-19 pandemic has led to rapid acceleration of vaccine development. According to the World Health Organization, 10 vaccine candidates are currently in different clinical phases, and 123 vaccines are being evaluated in preclinical models [3]. Current management of COVID-19 includes infection prevention and supportive care, such as oxygen supplementation and maintenance of a continuous positive airway pressure [4]. Preventative strategies, such as face mask–wearing, help reduce respiratory transmission of COVID-19. The World Health Organization recommends the use of face masks among those who provide care to a person with suspected COVID-19 [5].

Protection against COVID-19 among health care workers is key to providing effective care; however, latest studies from China have reported a high number of adverse reactions caused by personal protective equipment (PPE), specifically surgical and N95 face masks. In a sample of 542 health care workers, 97% were found to have facial or hand dermatoses [6-10]. Despite published guidelines recommending to limit the time of wearing N95 masks to 2 hours, health care workers often wear masks for much longer periods [6]. The consequences of prolonged mask-wearing include the following: pressure-related injuries, various dermatoses, skin dryness, skin erythema, acne, eczema, urticaria, rosacea, secondary infections, nasal bridge ulceration, and exacerbation of known skin disorders [11,12]. The objective of this study is to review the emerging literature on newly emerging dermatological conditions as a result of occupational mask-wearing.

## Methods

A narrative literature review was performed between May 1 and 29, 2020, in order to identify studies that evaluated the relationship between mask-wearing during the COVID-19 pandemic and the increase in the prevalence of certain dermatological conditions. Key search terms used herein included “covid mask dermatology,” “covid dermatological damage,” “covid mask skin,” “covid N95 mask damage,” and “covid mask skin damage.” The term “covid” was also replaced by “coronavirus,” “nCoV,” and “SARSCoV2” to increase the number of studies churned by the search. PubMed was the main electronic database that was searched, with search supplementation from Google Scholar and ResearchGate to identify missing articles. On eliminating duplicated searches, we found that the majority of studies found on Google Scholar and ResearchGate were duplicates of those found on PubMed. Qualitative results were obtained by comparing and summarizing results from all relevant and emerging studies by an independent researcher.

The inclusion criteria set for this study were vast owing to the limited literature sources and the novel exploratory design of the literature review. All articles included within the review explored new cases of dermatological damage caused by occupational use of PPE, focusing specifically on the damage caused by surgical and N95 masks, during the COVID-19 pandemic. Articles were sought from multiple institutions worldwide. Moreover, all articles focused on the effects of skin damage among health care workers; by definition, health care workers include physicians, nurses, health care assistants, pharmacists, students, therapists, and laboratory staff. Study designs included within the literature review were randomized controlled trials, cohort studies, and case studies. To further expand the breadth of the literature assessed, the review also accepted letters to the editors, commentaries, editorials, and perspectives. Articles that were case reports of individual patients’ dermatological findings in relation to COVID-19 were excluded from the study. Additionally, dermatological findings from previous pandemics including severe acute respiratory syndrome, Middle East respiratory syndrome, and Ebola were excluded.

On searching the aforementioned electronic databases, a total of 287 articles were found (PubMed: n=185, Google Scholar: n=67, and ResearchGate: n=35). After eliminating duplicate searches, 203 articles were screened by title and abstract, and a total of 120 articles were analyzed in full text. After full-text analysis, 40 articles, which satisfied the inclusion criteria, were included in the study. Through manual searches of the included articles’ reference lists, an additional 7 articles were identified. Prominent findings from the literature review are presented in a table under the following headings: dermatological diagnosis, causes, and management.

## Results

A total of 287 articles were found from various searches. After the elimination of duplicated articles, the title and abstract of 203 articles were screened. Of these, 120 articles were deemed appropriate for full-text screening, of which 40 articles met the inclusion criteria. Manual review of their reference lists yielded an additional 7 articles.

The findings of these 47 papers reviewed in this study are summarized in Table 1. The table documents reported dermatological diagnoses resulting from prolonged mask-wearing with their relevant causes and the management of conditions. Qualitative analysis of the included articles was conducted.

**Table 1:** A table summarizing the causes and management of dermatological conditions resulting from occupational mask-wearing.

Dermatological diagnosis	Causes	Management
Pressure-related injuries	Facial protective equipment, such as masks, place a significant amount of pressure on different facial areas, most notably the nasal bridge [13]. This can often cause numerous injuries at different facial points [14]. Pressure, friction and the hyperhydration effect caused by masks and goggles often result in skin indentation, mechanical skin damage, and epidermal barrier breakdown [12,15]. N95 masks specifically have increased air impermeability and a higher local pressure, increasing the risk of dermatological symptoms [7]. Risk factors for pressure damage are the following: prolonged wearing of personal protective equipment (PPE) [14-17], repeated wearing of PPE [15], use of grade 3 PPE [14], joint use of masks and goggles [7], high humidity [15], and heavy sweating [14]. Conflicting findings based on the relevance of gender and pressure-related injuries were reported in several studies [7,14,17].	Measures to reduce pressure-related injuries include the following: education of health care workers [7,17], wearing a properly fitted mask to minimize friction at specific points [7,11], regular moisturizing before and after the use of facial protective equipment for skin barrier repair [6,7,11,16], and limiting the time spent using a mask; published guidelines suggest limiting mask-wearing to 2 hours [6,11,13,16]. Pressure-related injuries that are progressive or cause discomfort to the user may be relieved by the use of a hydrocolloid dressing [6,16-18]. Hydrocolloid dressings are composed of water, sodium polyacrylate, cellulose gum, and sodium hyaluronate; these components serve as a cushion for soft tissue, thus reducing pressure and retaining skin moisture [18]. Dong et al [18] conducted a study to observe if hydrogel patches relieve skin damage in 19 health care workers; they reported that using hydrogel patches resulted in a lower mean score for skin reactions (3.47, SD 1.39, compared to nonuse scores of 13.32, SD 2.06), demonstrating that hydrogel patches are able to reduce the emergence and severity of skin damage [18]. Furthermore, they reported that the use of hydrogel patches reduced skin indentation as well as pain [18]. Conflicting evidence was reported on whether hydrocolloid dressings impacted the seal of facial masks [6,16,19]. High levels of humidity are reportedly a predisposing factor to skin barrier damage [15]; to reduce humidity levels, it is recommended to line masks with a paper towel or gauze [15].
Irritant contact dermatitis (ICD)	ICD is a common problem reported by health care workers [7,17,20]; symptoms include burning, itching, and stinging [11]. Formaldehyde, a material used in both surgical and N95 masks, has been recognized to be a frequent contact sensitizer for many people [7,16]. Acute and chronic dermatitis may be a result of skin and mucus membrane damage [11]. Facial protective equipment may induce ICD through occlusion and friction from the mask and the hyper-hydration effect of PPE; in turn, this breaks down the epidermal barrier of the skin [20]. Factors that predispose individuals to ICD include the following: increased moisture, warm environments, occlusion due to local pressure, and friction [7,21].	Protective measures include the following: ensuring the proper fit of the mask, labeling of contact sensitizers on face masks [17], cooling the skin by ensuring adequate air conditioning at the site, and wiping skin to remove sweat at appropriate times [20]. Staff should limit the duration of mask-wearing by having rotating shifts and regular mask-free breaks [20]. Furthermore, staying hydrated may also reduce symptoms of dermatitis [20]. Treatment of ICD includes the use of emollients before wearing masks [11,20,22]; emollients should be applied at least 30 minutes before wearing the mask to prevent damage to the mask [20]. Staff may also choose to line the mask with gauze to reduce the humidity [11]. For moderate to severe ICD, topical glucocorticoids may be recommended [11,20,22].
Allergic contact dermatitis (ACD)	Occupational ACD was also a common problem reported among health care workers [17]. ACD has a similar set of symptoms to ICD, which includes the following: pruritus, burning sensations, facial and periocular erythema, and subtle eczematous lesions [12,17]. Aggravating factors that may induce ACD include the following: prolonged use of PPE [7], increased moisture from perspiration, occlusion effects from the mask [7,23], friction [7,23], atopic predisposition [7], and contact sensitizers including formaldehyde [17,23]. Maliyar et al [23] reported that 22.8% of the population is sensitive to formaldehyde.	The gold standard for the diagnosis of ACD is patch testing [7]. The treatment for ACD is similar to that recommended for ICD. It is important to ensure correct fitting of PPE [23], the use of facial moisturizers before and after using PPE [11,23], the avoidance of facial cleansing with overheated water, 75% ethanol, or a facial cleanser [11], and the use of hydrogel dressings on damaged skin [23]. Layers of gauze inside the mask may be used to reduce moisture effects within the mask [11]. For mild dermatitis, the use of emollients is adequate; if the dermatitis progresses, topical glucocorticoid ointments may be used [11].
Retroauricular dermatitis	Retroauricular dermatitis is characterized by itching, redness, and scaling within the auricular region [24]. Ear pressure through the use of ear-hook masks is a reported cause of this type of dermatitis [15].	Recommendations to reduce dermatitis and ear pain include the following: the use of strings or hairpins to lengthen the ear-hook string [15] help reduce the tightness of masks [25]. Jiang et al [26] explored the use of a plastic handle to reduce ear pressure exerted by N95 masks; the advantage of this method was the simplicity of the idea and the increase in comfort on using the masks.
Skin lesions	Pei et al [27] reported that 73.1% of participants developed skin lesions due to PPE in a cohort of 484 health care workers. Skin lesions included the following: erythema, prurigo, blisters, rhagades, papule, oedema, exudation, crusting, and lichenification [12,27]. The most common sites were the nasal bridge as well as the cheeks and forehead [12]. Factors attributed to skin lesions included the following: higher grades of PPE, higher working frequency within PPE, and prolonged use of PPE [9,25,27].	Measures to reduce the incidence of skin lesions include the following: wearing the mask correctly, taking mask-free breaks, and frequently replacing of protective gear [25].
Skin dryness	In a sample of 542 participants in China, skin dryness was the most commonly reported symptom (70.3%) [9]. Closed humid environments, such as those resulting from breathing in masks and the use of PPE [9], result in skin barrier dysfunction [11,28]. Skin barrier dysfunction may consequently lead to skin dryness and scaling [11].	Management of skin dryness involves the use of high-potency moisturizers before and after PPE use [11].
Skin erythema	Hua et al [29] reported increased erythema following PPE use; erythema results from cutaneous blood vessel dilation and increased blood supply to the skin [29]. Although this may be a normal reaction to heat and pressure, long-lasting erythema may be a sign of inflammation [29]. Significant differences between the use of N95 masks and surgical masks have been reported; N95 masks reportedly increase the facial temperature of the user and are perceived to be more uncomfortable [30]. Factors potentially causing skin erythema include long hours and prolonged mask-wearing [20]. Campbell et al [31] reported that skin erythema may progress to miliaria owing to the associations of immobility and humidity through prolonged mask-wearing.	Measures to reduce skin erythema include the following: limiting shift length [20], having mask-free breaks [20], and using a surgical mask rather than an N95 mask when appropriate [30].
Skin injury due to the use of disinfectants	Skin injury due to the use of disinfectants may result in ICD [32] and ACD [13]. Excessive stress among health care workers because of working with patients with COVID-19 may increase the frequency and duration of skin cleansing, which disrupts the skin barrier and inevitably leads to skin damage [13].	Although it is important to clean the face using soap-based cleansers after contact with patients with COVID-19 owing to the high risk of disease transmission, health care workers should be wary of excessive washing and the repeated application of disinfectants to the skin [13].
Secondary infections	Skin and mucous membrane injury, through the disruption of the epidermal barrier, may lead to secondary infections [11]. Factors aggravating membrane injury include the following: prolonged mask-wearing resulting in a closed environment, compression, friction, and humidity [33].	Avoidance of PPE and the use of antihistamines and antibiotics are recommended for the treatment of secondary infections [13,33]. To prevent secondary infections, it is important to stop water from entering damaged skin; this can be done using waterproof plasters [33].
Acne vulgaris	Flares of acne have been reported to result from the use of facial protective equipment; this is thought to be due to increased temperature and humidity caused by the mask [34]. High temperatures as well as high humidity facilitate the progression of acne due to bacterial proliferation and the portal occlusive effect of skin hydration and irritation to the upper parts of the pilosebaceous duct; in turn, this causes swelling of the epidermal keratinocytes, leading to acute blockage of the skin barrier [11,34]. Other underlying mechanisms potentially include pressure and friction [11]. Interestingly, Han et al [34] observed no correlation between acne severity and prolonged mask-wearing. Signs of acne include comedones, papules on the cheeks and nose, as well as nodules or cysts on the forehead, submaxillary, and neck region [34].	The management of acne vulgaris includes the following: liberal use of moisturizers before and after using facial protective equipment, topical antibiotic creams for mild papules and pustules, as well as topical retinoid creams for blackheads and whiteheads [11]. Cases of severe acne vulgaris should be referred to a dermatologist [11].
Eczema	Navarro‐Triviño et al [35] found eczema to be one of the most frequently reported skin diseases associated with PPE use. The risk of eczema increased with continuous use of masks and protective glasses [32,35] as a result of increased heat owing to the closed environment, and increased stress [28].	Use of topical glucocorticoid creams or ointments is suggested for eczematous skin changes [13].
Rosacea	Rosacea has been frequently reported in association with PPE use [35]. Increased heat and stress is linked to the exacerbation of rosacea [28]. Prolonged PPE use is a risk factor for developing rosacea [32].	N/A^a^
Urticaria	Urticaria of the face has been linked to the resulting vertical pressure of facial protective equipment [11]. Risk factors include the following: prolonged wearing of protective equipment and excessive personal hygiene [12].	Preventative measures include the use of correctly fitted protective equipment and antihistamines [11].
Impetigo	Yu et al [36] documented a case of impetigo due to occupational goggle-mask–wearing during the pandemic [36]. Increased humidity, skin trauma, and malnutrition can increase the skin’s vulnerability to infection and create a moist occlusive environment, allowing *Staphylococcus aureus* to grow and infect the damaged skin [36].	Management of this condition included rest away from PPE and the application of topical 2% fusidic acid cream twice daily [36].
Nasal bridge ulceration	Owing to occupational use of PPE, the nasal bridge was reported to be damaged in 83.1% of health care workers [37]. Pressure, friction, and the hyperhydration effect are known risk factors for ulceration [12,15.]	Hydrocolloid dressings may be of use to successfully treat nasal bridge ulceration [37].
Exacerbations of known skin disease	Flares of pre-existing dermatoses have been reported to result from PPE use [17,28,32]. Stress, due to the pandemic, has been linked to the aggravation of skin conditions such as psoriasis, eczema, atopy, and neurodermatitis [28,32].	Zheng et al [32] questioned the use of psychological counseling to reduce the stress experienced by health care workers in order to reduce exacerbations of skin diseases.

^a^N/A: not applicable.

## Discussion

### Principal Findings

This preliminary and exploratory review documents the different dermatological conditions associated with occupational mask-wearing, by causes and management, among health care workers during the COVID-19 pandemic from the existing literature (in May 2020) (Table 1).

### Personal Perspectives of Health Care Workers in Relation to Dermatological Problems

The literature reveals a high number of health care workers who are affected by skin damage; in a sample of 546 individuals, 526 (97%) staff members reported negative skin consequences as a result of PPE use [6-10]. Symptoms of skin barrier damage, as reported by health care professionals, include burning, itching, and stinging [11]. The most common site of skin damage was the nasal bridge, and this occurred in 83.1% of health care workers [6-8,13].

Pei et al [27] conducted a study involving 484 health care workers and reported that 73.1% experienced various skin lesions including the following: erythema (38.8%), prurigo (22.9%), blisters (13.8%), rhagades (13.6%), papules (12.8%), exudation (6.8%), and lichenification.

Facial erythema was reported at varying rates; Pei et al [27] reported that 38.8% of health care workers experienced erythema, whereas Balato et al [38] found erythema rates to be higher at 60.4%. Singh et al [20] categorized two varying forms of erythema: whole face erythema (linked to prolonged hours) and lip lick erythema (linked to constant licking of lips from excessive thirst and fluid restriction). Scarano et al [30] investigated facial skin temperature in relation to occupational mask-wearing and reported a significant difference between surgical and N95 masks with regard to humidity, heat, breathing difficulty, and discomfort. Erythema, as a result of increased warmth, may cause health care workers to alter the position of their mask using contaminated hands, which may increase the risk of self-infection with COVID-19 [30].

Furthermore, skin papules have been reported to result from mask-wearing; papules are often a common sign of acne, alongside other symptoms such as comedones, nodules, and cysts [34]. Gheisari et al [21] reported that 35.5% of health care workers experienced acne as a consequence of occupational mask-wearing. Skin damage, such as an irritating pimples in the case of acne, may cause health care workers to repeatedly touch their face, thus increasing the risk of infection [34,39].

Iatrogenic skin damage, resulting from allergic and irritant contact dermatitis, is associated with occupational mask-wearing. In a sample of 14 health care workers, 35.7% of participants developed irritant contact dermatitis, and 28.6% of participants developed allergic contact dermatitis, which was associated with facial masks [24].

Another common complaint among health care workers concerned pressure-related injuries. Jiang et al [14] conducted a cross-sectional study, incorporating the views of 4308 health care workers and reported that 42.8% of respondents had skin injuries resulting from pressure (95% CI 41.30-44.30). Moreover, health care workers develop multiple skin lesions across different areas of the face; the disruption of the epidermal skin barrier across multiple sites may increase the risk of contracting COVID-19 among health care workers [14].

Lastly, Szepietowski et al [40] investigated the prevalence of pruritus among health care workers as a result of mask-wearing. From among 1393 participants, 273 (19.6%) reported an itching sensation. Higher incidences of pruritus have been reported in studies from Singapore and China; in Singapore, 51.4% of health care workers developed a facial itch [24], as opposed to 61.8% in China [27]. The risk of pruritus was further increased among those with an atopic predisposition, facial dermatoses, and prolonged PPE use [40]. Moreover, it was found that the sensation of pruritus caused health care workers to itch and touch their mask, reducing its protectiveness against COVID-19 [40].

### Recommendations to Reduce the Adverse Effects Associated With Facial Masks

Despite evidence regarding the vast number of dermatological conditions resulting from mask-wearing, limited evidence is available on the occupational management of these problems. One study advocated the use of virtual occupational health checks to prevent serious skin damage among health care workers [41]. The virtual clinic was led by nurses who advised health care workers on protective self-care and skin care measures and triaged moderate and severe skin disorders to dermatologists if needed [42].

Pressure-related injuries associated with occupational mask-wearing has been common. Letters to the editor, written by multiple working health care professionals throughout the current pandemic, have highlighted the immense discomfort and pressure damage faced by staff on a regular basis [13,16,41]. Surprisingly, despite reports of discomfort and pressure damage, Jiang et al [14] reported that only 17.7% of health care professionals used prophylactic dressings and lotions. Hydrocolloid dressings have been suggested to reduce skin damage and improve comfort among health care workers who use facial masks [16]. A study by Dong et al [18] found that the use of hydrogel patches resulted in lower skin reaction mean scores (3.47, SD 1.39) compared to their nonuse (13.32, SD 2.06), demonstrating their ability to reduce the severity and incidence of skin damage. Furthermore, Payne [16] argued that a strip of hydrocolloid dressing over the area of pressure damage should not impair the mask seal and should be used by health care workers with pre-existing skin disease or those who wear masks for over 2 hours. Buckley et al [6] agreed with the use of hydrocolloid dressings; however, they recommended refit testing for staff members to ensure the seal was intact to prevent infection. In stark contrast, Yin et al [41] reported that hydrocolloid dressings may be harmful to the skin owing to the extreme stickiness of the dressing and the potential to rip away skin on removal. A recent study by Jiang et al [26] documented the use of a plastic handle on the N95 respirator to reduce pressure injuries to the ear and reduce mask adjustments made by health care workers. The advantages of using a plastic handle included improved comfort, intact mask seal, reduced risk of infection through a lower rate of mask adjustments, and easy disinfection of the handles [27]. Other methods to reduce pressure-related injuries include the following: education of health care professionals [7,38], use of a correctly fitted mask to minimize friction [7,11], regular moisturizing [6,7,11,16], and frequent mask-free breaks [6,11,13,16].

The alleviation of mental health conditions, such as stress and anxiety, within these unprecedented times also plays a key role in preventing skin damage. Li et al [33] reported that high mental stress may precipitate endocrine disorders such as acne through excessive secretion of androgens, which in turn stimulate excess sebum secretion from the sebaceous glands. This, along with mask-wearing, reduces local blood circulation and oxygen levels and causes occlusion of the sebaceous ducts in hair follicles [33]. Additional factors that exacerbate skin stress include high-intensity work, irregular eating habits, and poor rest [32]. Other studies further corroborated the findings of Li et al, linking stress to acne [28], dermatitis [28], and pre-existing skin disorders [28,32].

In addition, anxiety has proved to be a problem among health care workers. A study conducted in Malaysia [41] reported that several pressure-related injuries were self-inflicted by health care professionals owing to overtightening of their N95 masks. Although overtightening of the masks provided the staff with mental relief of improved protection, it consequently increased the risk of skin damage and inadvertently increased the risk of COVID-19 infection through disrupted skin [41]. Staff members responsible for providing care to patients with COVID-19 should be educated on the efficacy of masks to reduce overtightening as well as anxiety [41].

Furthermore, nonmodifiable risk factors, such as gender, influenced the progression of skin damage. One study [25] reported that rashes were more likely to affect women. Zuo et al [39] found that women had a lower threshold for reporting symptoms of skin damage. However, Gefen et al [43] surveyed 4308 health care workers and found that the male prevalence of pressure injuries was significant and 1.6-fold that among women. Possible theories for this finding include differences in the facial architecture between men and women [41]. Moreover, this result may demonstrate the need for gender-specific PPE to prevent skin damage among men [41].

### Limitations

Notable limitations of our study include the exploratory direction of the literature review; the available literature was restricted owing to the emerging nature of COVID-19 and lack of studies during the search period (May 1-29, 2020). Moreover, the data included within the review may be constrained by PubMed being the only legitimate scientific database being used herein; this may raise concerns that other important studies may have been missed. As a result of limitations arising from the aforementioned reasons, our literature review accepted all types of articles, which may have limited the applicability of the results to the broader population. Furthermore, numerous studies included within our literature review reported their findings on the basis of a small cohort, which may have decreased the reliability of the results. Furthermore, articles included in this literature review were from multiple institutions worldwide, most notably the United Kingdom and Asia. Difficulties, such as finding reliable translations of articles as well as a lack of literature from other countries, may also skew the results of this literature review.

### Conclusions

PPE has been invaluable throughout the COVID-19 pandemic; it has allowed health care workers to safely provide care to the most vulnerable individuals, with minimal risk. Masks have provided the main form of essential protection to the respiratory system against COVID-19; however, owing to the rapid global threat COVID-19 presents, it is clear that the risk of skin damage resulting from mask-wearing has not been considered. The effects of skin damage can be dangerous among health care workers; the risk of infection may be increased through disruptions in the skin barrier as well as self-contamination through mask adjustments. The highly contagious nature of SARS-CoV-2 increases the likelihood that protective measures may stay in place from this day forward; innovation and advancements in PPE need to be sought to protect the skin and to reduce the currently increasing incidence of dermatological conditions among health care workers.

## Abbreviations

ACD: allergic contact dermatitis
ICD: irritant contact dermatitis
PPE: personal protective equipment
